# Atrial fibrillation and risk of progressive heart failure in patients with preserved ejection fraction heart failure

**DOI:** 10.1002/ehf2.14004

**Published:** 2022-07-04

**Authors:** John Gierula, Charlotte A. Cole, Michael Drozd, Judith E. Lowry, Sam Straw, Thomas A. Slater, Maria F. Paton, Rowenna J. Byrom, Ellis Garland, Georgia Halliday, Sarah Winsor, Gemma K. Lyall, Karen Birch, Melanie McGinlay, Emma Sunley, Peter J. Grant, David H. Wessels, Elias M. Ketiar, Klaus K. Witte, Richard M. Cubbon, Mark T. Kearney

**Affiliations:** ^1^ Leeds Institute of Cardiovascular and Metabolic Medicine University of Leeds Leeds UK; ^2^ Leeds Teaching Hospitals NHS Trust Leeds UK; ^3^ Faculty of Biological Sciences University of Leeds Leeds UK; ^4^ Accelerated Enrollment Solutions Horsham PA USA; ^5^ University Clinic, RWTH Aachen DE USA

**Keywords:** Heart failure preserved ejection fraction, Progressive heart failure, Atrial fibrillation

## Abstract

**Aims:**

Understanding of the pathophysiology of progressive heart failure (HF) in patients with heart failure with preserved ejection fraction (HFpEF) is incomplete. We sought to identify factors differentially associated with risk of progressive HF death and hospitalization in patients with HFpEF compared with patients with HF and reduced ejection fraction (HFrEF).

**Methods and results:**

Prospective cohort study of patients newly referred to secondary care with suspicion of HF, based on symptoms and signs of HF and elevated natriuretic peptides (NP), followed up for a minimum of 6 years. HFpEF and HFrEF were diagnosed according to the 2016 European Society of Cardiology guidelines. Of 960 patients referred, 467 had HFpEF (49%), 311 had HFrEF (32%), and 182 (19%) had neither. Atrial fibrillation (AF) was found in 37% of patients with HFpEF and 34% with HFrEF. During 6 years follow‐up, 19% of HFrEF and 14% of HFpEF patients were hospitalized or died due to progressive HF, hazard ratio (HR) 0.67 (95% CI: 0.47–0.96; *P* = 0.028). AF was the only marker that was differentially associated with progressive HF death or hospitalization in patients with HFpEF HR 2.58 (95% CI: 1.59–4.21; *P* < 0.001) versus HFrEF HR 1.11 (95% CI: 0.65–1.89; *P* = 0.7).

**Conclusions:**

*De novo* patients diagnosed with HFrEF have greater risk of death or hospitalization due to progressive HF than patients with HFpEF. AF is associated with increased risk of progressive HF death or hospitalization in HFpEF but not HFrEF, raising the intriguing possibility that this may be a novel therapeutic target in this growing population.

## Introduction

Chronic heart failure (HF) is a leading cause of mortality and morbidity worldwide.^1^, ^2^ It is thought to develop because of conditions impacting negatively on left ventricular (LV) function, including ischaemic heart disease, hypertension and valvular heart disease. HF has traditionally been viewed as a failure of LV systolic function, with reduced LV ejection fraction (EF) used to define systolic dysfunction, assess prognosis, and select patients for therapeutic interventions.^3^ However, it is well established that HF can occur in the presence of LVEF in the normal range: this so‐called HF with preserved EF (HFpEF), now accounts for a substantial proportion of clinical cases of HF.^4^, ^5^

It is similarly well‐established that patients with HFrEF, after an initial insult to LV function and a period of stable symptoms can enter into a downward spiral of declining LV systolic function, characterized by fluid retention, symptomatic deterioration, hospitalization requiring intravenous loop diuretics, and premature death.^6^ Clinical trials of drugs targeting activation of the renin angiotensin aldosterone (RAAS) and sympathetic nervous system (SNS), shown to reduce risk of death and hospitalization due to progressive HF in patients with HFrEF, have not shown such favourable results in patients with HFpEF.^7^ The results of these trials, and the encouraging results from the recent EMPEROR‐preserved trial,^8^ suggest that some of the mechanisms leading to progressive HF in patients with HFpEF are shared and others may differ from patients with HFrEF, although studies have not yet addressed this fundamental question, nor have studies directly compared risk factors for progressive HF in unselected patients with a new diagnosis of HFpEF or HFrEF. Our aim was to explore a wide range of potential risk factors that are differentially associated with progressive heart failure outcomes in patients with HFpEF versus HFrEF.

## Methods

We performed a prospective cohort study of all patients referred to a secondary care specialist HF clinic, from a primary care catchment of over 750 000 people between 1 May 2012 and 1 May 2013, with suspicion of HF based upon clinical signs and symptoms of HF and elevated NT‐pro‐BNP. Upon arrival at the clinic, demographic details, medical history, height, weight, and medical therapy were recorded, and patients underwent clinical assessment. A venous blood sample was taken for measurements of full blood count, electrolyte concentrations, and assessment of renal and liver function. Blood pressure was taken (right arm recumbent), and 12‐lead electrocardiography and trans‐thoracic echocardiography were performed. Prognostic nutritional index (PNI), which assesses nutritional status and inflammatory/hepatic function based on clinical marker values using the following equation: 10 × serum albumin concentration in g/dL + 0.005 × total lymphocyte count per mm^3^,^9^ was calculated for each patient. Vital status data were collected using linked Hospital Episode Statistics and Office of National Statistics mortality data. The study complied with the Declaration of Helsinki and received S251 ethical approval (CAG 8‐03(PR1)/2013).

### Natriuretic peptides

NT‐pro‐BNP concentration was measured in samples taken in primary care using the Immulite 2000 assay (Siemens Healthcare Diagnostics, Camberley, UK) in the biochemistry laboratory at the Leeds Teaching Hospitals NHS Trust. The inter batch coefficient of variation was 8.9% at 350 pg/mL and 5.9% at 4100 pg/mL.

### Echocardiography

Two‐dimensional trans‐thoracic echocardiography was performed by senior cardiac sonographers (J. G., M. P., and J. E. L.) blinded to NT‐pro‐BNP measurements. Left ventricular (LV) dimensions, left ventricular ejection fraction (LVEF), LV mass, left atrial (LA), and LV Doppler measurements were calculated according to the American Society of Echocardiography (ASE) and European Association of Cardiovascular Imaging (EACI) guidelines,^10^ and LV mass and LA volume were indexed to body surface area.

### Electrocardiography

Standard 12‐lead ECGs were recorded at 25 mm/s and analysed by a senior cardiologist blinded to patient characteristics. Classification of atrial rhythm status. Patient's atrial rhythm status was determined by their ECG at the clinic visit. Duration of AF was determined by medical records, and patients with persistent or permanent AF were categorized as having AF.

### Patient classification

Patients were categorized using the European Society of Cardiology 2016 guidelines on the diagnosis of HFrEF or HFpEF.^11^ We did not divide patients with EF < 50% into mid‐range and reduced ejection fractions and instead included all patients with EF < 50% as HFrEF. Patients with signs and symptoms of heart failure, and NT‐proBNP >125 pg/mL and an LVEF <50% were classified as HFrEF, patients with signs and symptoms of heart failure, an NT‐proBNP >125 pg/mL and an LVEF >50% and relevant structural heart disease (left atrial volume index (LAVI) > 34 mL/m^2^ or a left ventricular mass index (LVMI) ≥ 115 g/m^2^ for men and ≥95 g/m^2^ for women) or diastolic dysfunction (E/e′ ≥ 13 or a mean e′ septal and lateral wall <9 cm/s) were classified as HFpEF. Patients with signs and symptoms of heart failure an NT‐proBNP >125 pg/mL and not meeting the ESC criteria of either HFrEF or HFpEF were classified as neither HFrEF nor HFpEF, their final diagnoses can be found in Supporting Information, *Table* *S1*.

### Classification of patient outcomes

Patient follow‐up continued for a minimum of 6 years in surviving participants. HF hospitalization was *a priori* defined, using patient records as a new onset or worsening of signs and symptoms of heart failure with evidence of fluid overload requiring at least 24 h overnight hospitalization and the use of intravenous diuretics,^12^ and progressive HF death was defined if death occurred after a documented period of symptomatic or hemodynamic deterioration.^13^, ^14^ The combined endpoint of progressive heart failure was determined as either first HF related hospitalization or HF/cardiac related death.

### Statistical analysis

All statistical analyses were performed using IBM SPSS statistics version 26 (IBM Corporation, Armonk, NY, USA). Normal distribution of data was confirmed using skewness tests. Continuous data are presented as mean ± standard deviation or median [interquartile range] if non‐normally distributed; categorical data are shown as percentage (number). Groups were compared using two‐sided Student's *t*‐tests or ANOVA for normally distributed continuous data, Mann–Whitney or Kruskal–Wallis tests for non‐normally distributed continuous data, and two‐sided Pearson *χ* ^2^ tests for categorical data. Survival of groups was compared with Kaplan–Meier curves and log‐rank tests, or Cox proportional hazards regression analysis, for which non‐normally distributed data were log10 or natural log transformed to achieve normality. To explore if the extent of association between specific covariates and the composite outcome of progressive HF death or hospitalization was statistically different between people with HFrEF and HFpEF, interaction terms were added to models. Statistical significance was defined as *P* < 0.05.

## Results

Between 1 May 2012 and 1 May 2013, 982 patients with suspected heart failure and NT‐proBNP >125 pg/mL were referred. Of these, 22 had insufficient quality echocardiographic images to assess cardiac structure and function and so 960 patients were included in this analysis.

### Patient characteristics

Of the 960 patients referred, HFpEF was the most common diagnosis (*n* = 467; 49%) followed by HFrEF (*n* = 311; 32%) and neither HFpEF/HFrEF (*n* = 182; 19%). As shown in *Table* *1*, patients with HFpEF were older than those with HFrEF, more often female, more likely to have a history of hypertension, and less likely to have a history of ischaemic heart disease than patients with HFrEF. As expected, patients with HFpEF had significant differences in LVEF, compared with patients with HFrEF, but all other echocardiographic variables were similar. The number of patients prescribed disease modifying medical therapy was typical for a population newly referred with suspicion of HF.

**Table 1:** Characteristics of patients presenting to secondary care based on the European Society of Cardiology guidelines for the diagnosis of heart failure with preserved ejection fraction (HFpEF) and heart failure with reduced ejection fraction (HFrEF)

	All (*n* = 778)	HFpEF (*n* = 467)	HFrEF (*n* = 311)	*P* value
**Demographics and previous medical history**				
Age (years)	83.0 ± 9.2	83.7 ± 8.6	82.0 ± 10.0	0.009
Male sex, *n* (%)	344 (44%)	163 (35%)	181 (58%)	<0.001
Body mass index (kg/m^2^)	23.9 ± 5.4	24.0 ± 5.4	23.8 ± 5.3	0.710
Ischaemic heart disease, *n* (%)	220 (28%)	105 (22%)	115 (37%)	<0.001
Diabetes mellitus, *n* (%)	215 (28%)	112 (24%)	103 (33%)	0.005
Hypertension, *n* (%)	529 (68%)	353 (76%)	176 (57%)	<0.001
Atrial fibrillation, *n* (%)	279 (36%)	172 (37%)	107 (34%)	0.490
Chronic obstructive pulmonary disease, *n* (%)	114 (15%)	64 (14%)	50 (16%)	0.359
**Echocardiographic and haemodynamic data**				
Heart rate (b.p.m.)	75.7 ± 17.1	73.6 ± 14.	78.9 ± 19.6	<0.001
Systolic blood pressure (mmHg)	140.6 ± 23.8	144.1 ± 23.4	135.4 ± 23.4	<0.001
Left ventricular ejection fraction, %	48.4 ± 11.9	56.1 ± 4.1	36.6 ± 10.0	<0.001
E/A ratio	0.78 [0.63–1.16]	0.80 [0.66–1.11]	0.76 [0.60–1.25]	0.520^a^
E/e′	14.0 [10.0–18.0]	13.6 [10.0–17.0]	14.0 [10.0–21.0]	0.290^a^
Left atrial volume index (mL/m^2^)	38.6 [29.2–50.5]	38.5 [29.6–49.0]	39.1 [29.1–51.4]	0.689^a^
**Laboratory data**				
NT‐proBNP (pg/mL)	1054 [510–2562]	845 [438–1707]	1634 [686–3836]	<0.001^a^
Sodium (mmol/L)	140.1 ± 3.6	140.3 ± 3.7	139.7 ± 3.5	0.024
Prognostic nutritional index	42.4 [40.1–44.4]	42.3 [40.1–44.5]	42.4 [40.0–44.3]	0.962^a^
Creatinine (μmol/L)	82.0 [69.0–104.0]	79.0 [66.0–102.0]	85.0 [73.0–109.3]	0.001^a^
eGFR (mL/min/1.73 m^2^)	65.2 ± 19.5	65.4 ± 19.4	64.7 ± 19.7	0.548
Haemoglobin (g/L)	128.7 ± 19.4	128.1 ± 19.2	129.6 ± 19.6	0.284
White cell count (10*9/L)	6.9 [5.7–8.3]	6.9 [5.7–8.3]	6.9 [5.9–8.6]	0.316^a^
Lymphocyte count (10*9/L)	1.5 [1.1–2.0]	1.5 [1.2–2.0]	1.5 [1.1–2.0]	0.120^a^
Neutrophil count (10*9/L)	4.4 [3.6–5.5]	4.4 [3.6–5.4]	4.6 [3.7–5.7]	0.149^a^
Eosinophil count (10*9/L)	0.14 [0.90]	0.15 [0.90–0.23]	0.14 [0.09–0.23]	0.947^a^
Bilirubin (μmol/L)	9.0 [6.0–13.0]	9.0 [6.0–12.0]	9.5 [6.0–14.0]	0.058^a^
**Alanine transaminase (ALT)**	19.0 [15.0–26.0]	18.0 [14.0–24.0]	20.0 [15.8–27.0]	0.002^a^
Alkaline phosphatase (IU/L)	201.0 [165.0–202.0]	196.0 [160.0–245.0]	205.0 [171.0–261.0]	0.014^a^
Albumin (g/L)	41.4 ± 3.5	41.3 ± 3.6	41.4 ± 3.4	0.814
**Medication**				
Beta‐blocker prescription, *n* (%)	432 (56%)	257 (55%)	175 (56%)	0.734
Bisoprolol equivalent dose (mg/day)	2.8 ± 3.4	2.9 ± 3.5	2.7 ± 3.3	0.259
ACEi or ARB prescription, *n* (%)	471 (61%)	274 (59%)	179 (63%)	0.192
Ramipril equivalent dose (mg/day)	3.3 ± 3.8	3.3 ± 3.9	3.3 ± 3.7	0.786
Loop diuretic prescription, *n* (%)	370 (48%)	199 (43%)	171 (55%)	0.001
Furosemide equivalent dose (mg/day)	0.0 [0.0–40.0]	0.0 [0.0–40.0]	20.0 [0.0–40.0]	<0.000^a^
Thiazide prescription, *n* (%)	62 (8%)	46 (10%)	16 (5%)	0.018
Digoxin prescription, *n* (%)	34 (4%)	14 (3%)	20 (6%)	0.022
Statin prescription, (%)	322 (41%)	177 (38%)	145 (47%)	0.016
Calcium channel blocker prescription, *n* (%)	157 (20%)	103 (22%)	54 (17%)	0.110

Data presented as mean ± SD, median [IQR], or *n* (%); eGFR, estimated glomerular filtration rate; E/A ratio, the ratio between early diastolic mitral inflow velocity and late diastolic mitral inflow velocity; E/e′, the ratio between early diastolic mitral inflow velocity and mitral annular early diastolic velocity; ACEi, angiotensin‐converting enzyme inhibitor; ARB, angiotensin receptor blocker.

^a^ Kruskal–Wallis test.

### Mortality

**Figure 1:**
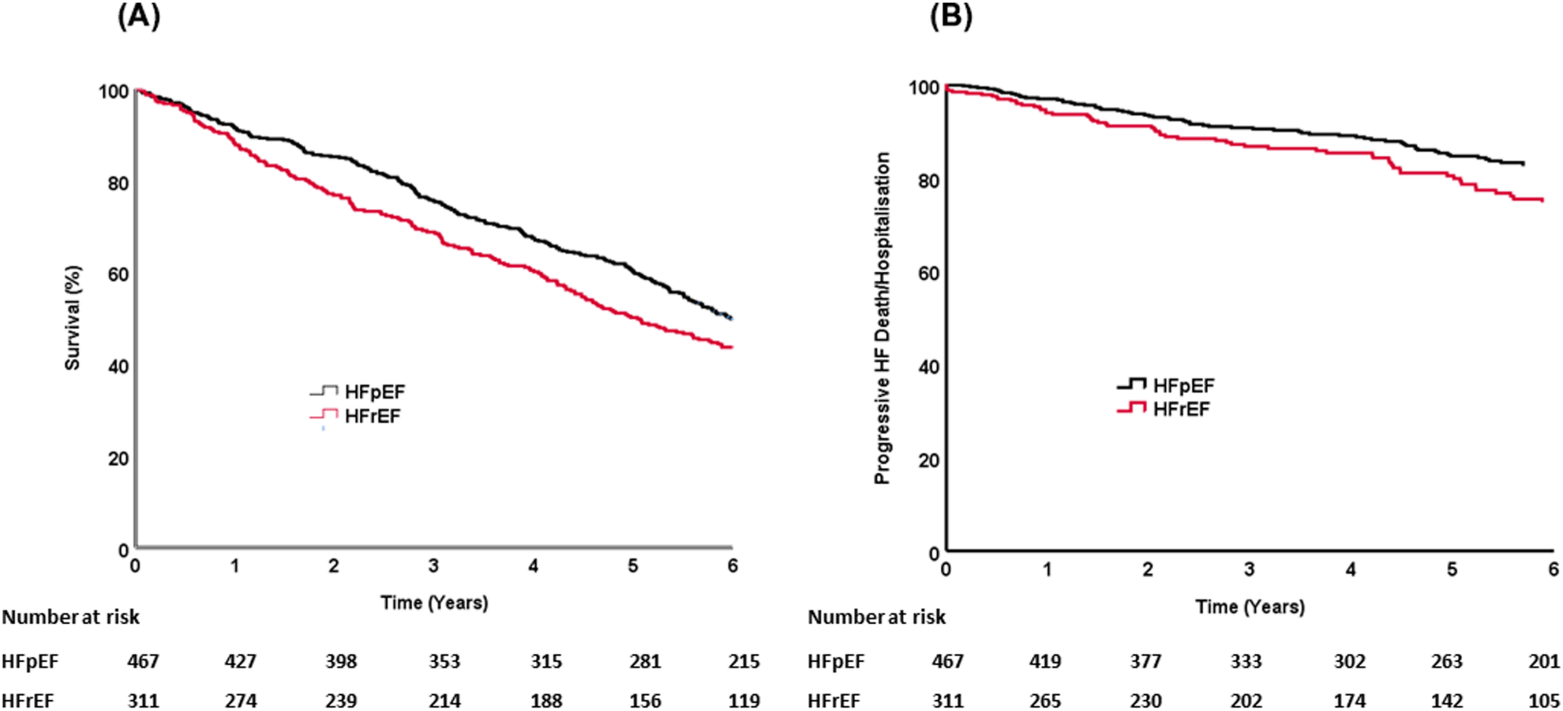
Long term outcomes of patients with either heart failure with reduced (HFrEF) or preserved (HFpEF) left ventricular ejection fraction. Survival curves of (*A*) total survival and (*B*) death or hospitalization from progressive heart failure over 6 years in patients presenting to secondary care with suspected heart failure classified according to European Society of Cardiology 2016 guidelines.

After a total of 3549 patient‐years follow‐up there were 497 deaths (52%). At 6 years, unadjusted survival rates were 46.0% (95% CI: 40.5–51.5%) in patients with HFpEF and 37.9% (95% CI: 33.4–42.4%) in patients with HFrEF (*P* = 0.016, log‐rank test. *Figure* *1* *A*). When adjusted for age and sex, patients with HFpEF had a better prognosis than those with HFrEF (hazard ratio 0.77; 95% CI: 0.63–0.93; *P* = 0.007).

### Factors associated with progressive heart failure hospitalization or death in heart failure with preserved ejection fraction or heart failure with reduced ejection fraction

**Figure 2:**
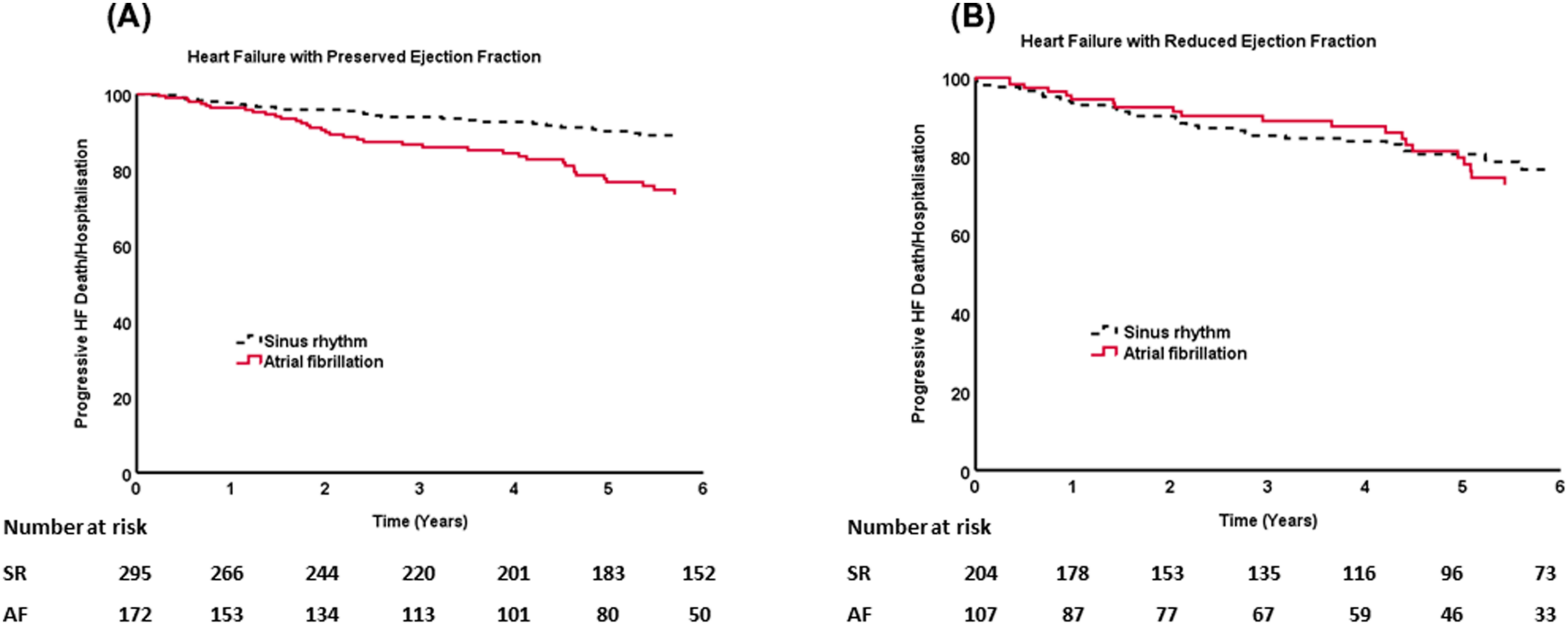
Long term outcomes of patients with either heart failure with reduced (HFrEF) or preserved (HFpEF) left ventricular ejection fraction by presence or absence of atrial fibrillation. Survival curves of death or hospitalization from progressive heart failure showing the adverse effect of atrial fibrillation in those with (*A*) heart failure with preserved ejection fraction but not in those with reduced ejection fraction (*B*).

During the follow‐up period there were 125 episodes of progressive heart failure hospitalization or death, 66 (53%) of these occurred in patients with HFpEF (of which 33 (50%) were due to progressive HF death and 33 (50%) were due to progressive HF hospitalization), and 59 (47%) events in those with HFrEF (of which 29 (49%) were attributable to progressive HF death and 30 (51%) due to progressive HF hospitalization). In patients with HFpEF, 14% died or were hospitalized due to progressive HF during the follow‐up period, compared with 19% of patients with HFrEF (*Figure* *1* *B*), age‐sex adjusted hazard ratio was 0.67 (95% CI: 0.47–0.96; *P* = 0.028). Univariate predictors of hospitalization or death from progressive heart failure in HFpEF and HFrEF are shown in *Table* *2*. Among a range of potential prognostic markers, the only factor differentially associated with risk of hospitalization or death due to progressive heart failure in HFpEF versus HFrEF was the presence of atrial fibrillation (p for interaction = 0.021), which persisted after adjusting for age and sex (*Table* *3*); survival curves for with and without atrial fibrillation in HFpEF or HFrEF are shown in *Figure* *2* *A,B*. We then examined characteristics of patients with HFpEF and HFrEF with and without atrial fibrillation (*Table* *4*). In patients with HFpEF ~36% had atrial fibrillation and in HFrEF ~34% (*P* = non‐significant). Patients with HFpEF and atrial fibrillation were older, more likely to be male, have a faster resting heart rate and lower systolic blood pressure, these differences were not apparent in the HFrEF group. We therefore performed further analysis to account for the potential influence of these factors in the interaction between atrial fibrillation and HFpEF in association with progressive heart failure adverse outcomes. After adjusting for age, sex, heart rate and systolic blood pressure, the interaction between atrial fibrillation and HFpEF persisted, suggesting that these factors did not contribute substantially to the interaction. We divided patients into tertiles of NT‐proBNP and this value, at baseline, predicts death and/or hospitalization due to progressive heart failure in patients with both HFpEF (log rank *P* < 0.001) and HFrEF (log rank *P* < 0.001) (Supporting Information, *Figure* *S1*), there was no significant difference between HFpEF and HFrEF, confirming our interaction analyses (*Table* *3*).

**Table 2:** Univariable hazard of death/hospital admission for progressive heart failure in patients presenting to secondary care based on the European Society of Cardiology guidelines for the diagnosis of heart failure with preserved ejection fraction (HFpEF) and heart failure with reduced ejection fraction (HFrEF)

Characteristic	HFpEF		HFrEF		Interaction *P* value
	Progressive heart failure HR (95% CI)	*P*	Progressive heart failure HR (95% CI)	*P*	
Age (per year)	1.058 (1.024 to 1.093)	0.001	1.024 (0.996 to 1.052)	0.090	0.131
Male sex	1.115 (0.675 to 1.842)	0.670	1.486 (0.871 to 2.535)	0.146	0.437
Heart rate (per b.p.m.)	1.016 (1.000 to 1.032)	0.050	1.012 (1.000 to 1.024)	0.047	0.705
Systolic blood pressure (per mmHg)	0.993 (0.983 to 1.004)	0.236	0.995 (0.984 to 1.007)	0.410	0.849
eGFR (per x decrease)	0.981 (0.969 to 0.993)	0.003	0.979 (0.966 to 0.992)	0.002	0.803
NT‐proBNP (per 10‐fold increase)	4.826 (2.947 to 7.904)	<0.000	3.366 (2.058 to 5.504)	<0.000	0.341
Ischaemic heart disease	1.194 (0.688 to 2.073)	0.529	1.300 (0.776 to 2.180)	0.319	0.831
Diabetes mellitus	1.150 (0.663 to 1.998)	0.619	1.414 (0.838 to 2.387)	0.195	0.597
Hypertension	0.832 (0.484 to 1.431)	0.507	1.256 (0.744 to 2.119)	0.394	0.284
Atrial fibrillation	2.584 (1.585 to 4.214)	<0.000	1.107 (0.650 to 1.887)	0.708	0.021
Beta‐blocker prescription	0.933 (0.575 to 1.514)	0.779	1.161 (0.680 to 1.981)	0.584	0.560
ACE/ARB prescription	0.849 (0.522 to 1.381)	0.511	1.611 (0.896 to 2.895)	0.111	0.102
Loop diuretic prescription	2.023 (1.245 to 3.287)	0.004	1.945 (0.770 to 4.912)	0.159	0.779

eGFR, estimated glomerular filtration rate; ACEi, angiotensin‐converting enzyme inhibitor; ARB, angiotensin receptor blocker.

**Table 3:** Absolute and adjusted hazard of hospitalization or death due to progressive heart failure in HFpEF and HFrEF due to atrial fibrillation

	Progressive heart failure HR (95% CI)	*P*	Progressive heart failure HR (95% CI)	*P*
Atrial fibrillation	2.584 (1.585 to 4.214)	<0.001	1.107 (0.650 to 1.887)	0.708
Atrial fibrillation, age	2.427 (1.487 to 3.959)	0.002	1.036 (0.605 to 1.774)	0.897
Atrial fibrillation, age, sex	2.420 (1.468 to 3.992)	0.001	1.012 (0.591 to 1.733)	0.965

**Table 4:** Characteristics of patients presenting to secondary care based on the European Society of Cardiology guidelines for the diagnosis of heart failure with preserved ejection fraction (HFpEF) and heart failure with reduced ejection fraction (HFrEF) with and without atrial fibrillation

	HFpEF *n* = 467		*P* value	HFrEF n = 311		*P* value
	Atrial fibrillation (*n* = 172)	Sinus rhythm (*n* = 295)		Atrial fibrillation (*N* = 107)	Sinus rhythm (*N* = 204)	
**Demographics and previous medical history**						
Age (years)	85.1 ± 7.6	83.0 ± 9.1	0.011	83.8 ± 8.7	81.0 ± 10.5	0.018
Male sex	80 (47%)	83 (28%)	0.000	66 (62%)	115 (56%)	0.367
Body mass index (kg/m^2^)	24.1 ± 5.9	23.9 ± 5.2	0.713	24.4 ± 5.1	23.5 ± 5.4	0.235
Ischaemic heart disease	42 (24%)	63 (21%)	0.444	33 (31%)	82 (40%)	0.104
Diabetes mellitus	40 (23%)	72 (22%)	0.779	41 (38%)	62 (30%)	0.158
Hypertension	129 (75%)	224 (76%)	0.821	60 (56%)	116 (57%)	0.894
Chronic obstructive pulmonary disease	22 (13%)	42 (14%)	0.661	11 (10%)	39 (19%)	0.044
**Echocardiographic and haemodynamic data**						
Left ventricular ejection fraction, %	55.4 ± 3.6	56.5 ± 4.3	0.006	37.2 ± 8.9	36.3 ± 10.5	0.456
Heart rate (b.p.m.)	78.6 ± 16.5	70.5 ± 12.7	0.000	81.0 ± 23.2	77.8 ± 17.5	0.192
Systolic blood pressure (mmHg)	139.2 ± 20.7	147.0 ± 24.5	0.001	134.4 ± 25.4	136.0 ± 22.4	0.595
Diastolic blood pressure (mmHg)	75.0 ± 12.2	73.2 ± 11.8	0.149	74.0 ± 14.1	72.3 ± 12.2	0.275
**Laboratory data**						
NT‐proBNP (pg/mL)	1533 [885–2743]	587 [308–1084]	0.000^a^	2736 [1604–4771]	1157 [518–2879]	0.000^a^
Sodium (mmol/L)	140.4 ± 3.7	140.3 ± 3.7	0.776	140.0 ± 3.3	139.6 ± 3.6	0.261
Creatinine (μmol/L)	83.0 [71.0–100.0]	78 [65–104]	0.143^a^	89 [74–111]	83.0 [72.0–109.0]	0.403^a^
eGFR (mL/min/1.73 m^2^)	66.4 ± 18.4	65.0 ± 20.0	0.458	63.8 ± 18.7	65.1 ± 20.2	0.567
Haemoglobin (g/L)	130.2 ± 18.9	126.9 ± 19.2	0.076	129.5 ± 17.8	129.7 ± 20.6	0.954
Prognostic nutritional index	42.4 [40.3–44.5]	42.3 [39.9–44.4]	0.349^a^	42.2 [39.5–44.3]	42.6 [40.2–44.4]	0.180^a^
White cell count (10*9/L)	6.9 [5.8–8.3]	6.9 [5.6–8.1]	0.467^a^	6.7 [5.7–7.8]	7.1 [6.0–9.0]	0.046^a^
Lymphocyte count (10*9/L)	1.5 [1.2–2.0]	1.6 [1.1–2.0]	0.698^a^	1.3 [1.0–1.7]	1.6 [1.1–2.1]	0.002^a^
Neutrophil count (10*9/L)	4.5 [3.7–5.5]	4.3 [3.5–5.4]	0.209^a^	4.4 [3.7–5.4]	4.6 [3.7–5.7]	0.357^a^
Eosinophil count (10*9/L)	0.14 [0.09–0.23]	0.15 [0.09–0.23]	0.962^a^	0.16 [0.08–0.23]	0.14 [0.09–0.23]	0.671^a^
Bilirubin (μmol/L)	10.0 [8.0–15.0]	8.0 [6.0–11.0]	0.000^a^	12.0 [8.0–16.0]	8.0 [6.0–12.0]	0.000^a^
**Alanine transaminase (IU/L)**	18.0 [14.0–24.0]	18.0 [14.5–23.5]	0.891^a^	21 [16–26]	20.0 [15.0–28.0]	0.829^a^
Alkaline phosphatase (IU/L)	195 [166–250]	197 [158–243]	0.492^a^	212 [170–260]	204 [173–262]	0.998^a^
Albumin (g/L)	41.5 ± 3.6	41.3 ± 3.6	0.486	41.2 ± 3.6	41.5 ± 3.2	0.393
**Medication**						
Beta‐blocker prescription	107 (62%)	150 (51%)	0.017	74 (69%)	101 (50%)	0.001
Bisoprolol equivalent dose (mg/day)	3.1 ± 3.4	2.8 ± 3.6	0.364	3.4 ± 3.4	2.3 ± 3.2	0.003
ACEi or ARB prescription	105 (61%)	169 (57%)	0.426	71 (66%)	126 (62%)	0.425
Ramipril equivalent dose (mg/day)	3.2 ± 3.6	3.4 ± 4.0	0.559	3.5 ± 3.6	3.1 ± 3.7	0.379
Loop diuretic prescription	91 (53%)	108 (37%)	0.001	67 (63%)	104 (51%)	0.050
Furosemide equivalent dose (mg/day)	20.0 [0.0–40.0]	0.0 [0.0–40.0]	<0.001^a^	40.0 [0.0–40.0]	20.0 [0.0–40.0]	0.013^a^
Aldosterone antagonist prescription	7 (4%)	4 (1%)	0.062	12 (11%)	9 (4%)	0.023
Thiazide prescription	14 (8%)	32 (11%)	0.344	6 (6%)	10 (5%)	0.789
Digoxin prescription	12 (7%)	2 (1%)	<0.001	18 (17%)	2 (1%)	<0.001
Statin prescription	66 (38%)	111 (38%)	0.873	50 (47%)	95 (47%)	0.979
Calcium channel blocker prescription	37 (22%)	66 (22%)	0.829	16 (15%)	38 (19%)	0.416

Data presented as mean ± SD, median [IQR], or *n* (%); eGFR, estimated glomerular filtration rate; ACEi, angiotensin‐converting enzyme inhibitor; ARB, angiotensin receptor blocker.

^a^ Kruskal–Wallis test.

## Discussion

Through exploiting a unique prospective cohort study specifically designed to examine prognostic markers in patients with new onset HFpEF or HFrEF we present novel findings that significantly add to our understanding of the pathophysiology of HFpEF. We show that patients with HFpEF have a reduced but important risk of hospitalization or death due to decompensated HF compared with patients with HFrEF, we also show that atrial fibrillation is the only marker of increased risk of hospitalization or death due to decompensated HF in patients with a new diagnosis of HFpEF distinct from patients diagnosed with HFrEF.

### Characteristics of patients with European Society of Cardiology defined heart failure with preserved ejection fraction

Consistent with our earlier reports of patients with HFpEF,^15^, ^16^ the ESC criteria for the diagnosis of patients with HFpEF identified a cohort which was older, predominantly female, had increased prevalence of hypertension with fewer patients having a history of ischaemic heart disease than patients with HFrEF. Previous studies assessing prognosis in patients with a diagnosis of HFpEF have predominantly relied on clinical signs and symptoms of CHF and a rudimentary dichotomy of LVEF of 50% or less to discriminate between HFpEF and HFrEF.^17^, ^18^, ^19^, ^20^ Many of these studies did not examine progressive HF death or hospitalization in these patients.

### Atrial fibrillation a predictor of progressive heart failure in patients with heart failure with preserved ejection fraction

In univariate analysis we found a number of shared predictors of risk of hospitalization or death due to decompensated HF in patients with HFpEF and HFrEF. The only marker of hospitalization or death due to decompensated HF that discriminated between patients with HFpEF and HFrEF was atrial fibrillation which was associated with a greater than two‐fold increase in risk of decompensated HF in patients with HFpEF. After adjustment for a number of variables including resting heart rate, sex, age and systolic blood pressure, atrial fibrillation remained differentially associated with progressive HF outcomes between patients with HFpEF and HFrEF, suggesting other factors may account for this intriguing observation.

### Atrial fibrillation and heart failure with preserved ejection fraction

Atrial fibrillation is a common co‐morbidity in people with HFpEF and may precede, coincide with, or develop following a diagnosis of HFpEF.^21^ In the present study, almost 40% of patients with HFpEF had atrial fibrillation at presentation. In longitudinal studies, the development of atrial fibrillation after the diagnosis of HFpEF has been shown to increase the risk of death.^22^ Zafrir *et al*. published similar findings to ours in demonstrating worse outcomes for patients with AF and HFpEF^23^; however, this dataset included people with acute and chronic HF and relied on clinical signs and symptoms of CHF and a rudimentary dichotomy of LVEF of >50% to diagnose HFpEF, whereas our dataset contains patients carefully categorized using the European Society of Cardiology 2016 guidelines on the diagnosis of HFrEF or HFpEF,^11^ and for the first time demonstrates that at first diagnosis of HF, despite a similar prevalence of atrial fibrillation as patients with HFrEF, patients with HFpEF and atrial fibrillation are more than twice as likely to die or be hospitalized urgently due to progressive heart failure.

Interestingly, data from the CASTLE‐AF randomized trial of AF ablation in patients with HFrEF and EF < 35% demonstrated that patients assigned to ablation had reduced incidence of death of HF hospitalization.^24^ Benefits were observed with a reduction in AF burden from 60% with medical therapy to 25% with ablation, suggesting that a reduction in the time spent in AF may be enough to provide clinical benefit. These data contrast with the results of our study; however, CASTLE‐AF had a relatively small number of participants, lack of blinded randomization and treatment allocation,^25^ and a relatively high number of patients dropped out or were lost to follow‐up. Sartipy *et al*. also present data at odds to ours, from the Swedish HF registry, in demonstrating adverse outcomes in patients with HFrEF and AF.^26^ However, hospitalized patients accounted for 64% of the patients recruited into the original Swedish registry,^27^ suggesting a more unstable population than our ambulatory cohort.

HFpEF and AF share similar risk factors and pathophysiological mechanisms,^28^ and while our data do not identify mechanisms underpinning this relationship, possibilities emerge from previous studies. One possibility is that HFpEF may be a result of a systemic disorder, which exerts a deleterious influence on the ventricle as well as on the atria.^29^ A second possibility is that changes in left atrial geometry are central to the pathogenesis of atrial fibrillation induced progressive heart failure in patients with HFpEF.^30^ Atrial involvement in HFpEF is well recognized: disadvantageous remodelling of the left atrium, and an excess of incident atrial fibrillation is consistently observed in patients with HFpEF.^22^, ^30^ Recent studies suggest that left atrial function and remodelling are independently associated with the onset of HF in the asymptomatic healthy population.^31^ Sanchis *et al*. reported that up to 45% of patients presenting with new‐onset symptoms to a dedicated HF clinic had left atrial dysfunction as the unique underlying mechanism of their HF symptoms, further supporting left atrial dysfunction as a potential driver of the HFpEF syndrome and a key pathogenic factor in its progression.^32^ In addition to atrial geometry and function, reduced left ventricular filling due to the lack of ‘atrial kick’ associated with AF might be particularly important in patients with HFpEF, due to the elevated filling pressures and impaired ventricular relaxation experienced by these patients.^33^

The strong clinical and epidemiological affinity of AF and HFpEF supports the potential of a common mechanistic substrate for the two diseases, inflammatory and fibrotic biomarkers predict AF and HFpEF and metabolic disorders have been linked to growth and inflammatory effects of epicardial adipose tissue.^22^ The results from the EMPORER‐Preserved trial,^8^ and the post‐hoc analysis of the TOPCAT trial^34^ raise the opportunity to learn how sodium‐glucose cotransporter 2 (SGLT2) inhibitors and aldosterone antagonists could influence these common mechanisms and impact on the deleterious AF/HFpEF relationship.

To our knowledge, ours is the first study to identify a distinct and potentially treatable baseline clinical feature that is linked to a specific outcome in patients with HFpEF, thereby raising the intriguing possibility that electrical or pharmacological treatment of atrial fibrillation aiming for sinus rhythm in patients with HFpEF has the potential to slow disease progression.

### Strengths and limitations of current study

This report has several strengths compared with earlier work in the field. While our own work,^15^, ^16^ and that of others,^17^, ^18^, ^19^, ^20^ confirms that HFpEF per se has a more favourable prognosis than HFrEF, a strength of our report is the unselected nature of the cohort studied resulting in a mean age of over 83 years for HFpEF patients attending the clinic from a large and diverse adult population, hence being truly representative of patients now presenting on a day‐to‐day basis. A second strength is comprehensive assessment of mode of death and hospitalization, providing a deeper understanding of the natural history of HFpEF. Some limitations need to be highlighted. We did not collect change in medical therapy, change in atrial rhythm status or imaging data during the follow‐up period, limiting our ability to relate any change in these characteristics to outcome data. Our study, being single centre may limit generalization; however, the diverse characteristics of the area served by our centre recently described by ourselves,^35^ mitigates against this potential weakness. We did not examine LV function invasively so our categorization of HFpEF relied on non‐invasive assessment of clinical status. The observational nature of the study, whilst opening new avenues for investigation, mean our insights into mechanisms of disease aetiology are hypothesis generating.

## Conclusions

HFpEF is a growing healthcare problem associated with significant morbidity and mortality. The mechanisms underlying the development and complications of HFpEF are poorly understood. Our dataset demonstrates that patients with HFpEF have reduced risk of progressive HF than patients with HFrEF. The critical finding that atrial fibrillation may drive the progression of disease in patients with HFpEF provides a platform to develop and evaluate treatments targeting atrial fibrillation for the burgeoning group of patients suffering from HFpEF.

## Conflict of interest

No authors have any conflicts in relation to the current subject matter.

## Funding

John Gierula and Maria Paton are supported by The National Institute of Health Research. Michael Drozd, Sam Straw, Thomas Slater, and Mark Kearney are supported by the British Heart Foundation.

## Supporting information


**Table S1.** Diagnoses of patients presenting to secondary care with neither European Society of Cardiology guidelines based diagnosis of heart failure with preserved ejection fraction (HFpEF) or heart failure with reduced ejection fraction (HFrEF).

**Figure S1.** Long term outcomes of patients with either heart failure with reduced (HFrEF) or preserved (HFpEF) left ventricular ejection fraction by tertiles of NTproBNP.
Survival curves of death or hospitalization from progressive heart failure showing for tertiles of NTproBNP in those with (A) heart failure with preserved ejection fraction and (B) heart failure with reduced ejection fraction.
