# Supplementary material for: Atrial fibrillation and risk of progressive heart failure in patients with preserved ejection fraction heart failure
Source: ESC Heart Fail. 2022 Jul 4;9(5):3254–63. doi: 10.1002/ehf2.14004 (PMC9715884; doi:10.1002/ehf2.14004)
Supplement: Supplementary file 1 — Table S1. Diagnoses of patients presenting to secondary care with neither European Society of Cardiology guidelines based diagnosis of heart failure with preserved ejection fraction (HFpEF) or heart failure with reduced ejection fraction (HFrEF). [file EHF2-9-3254-s001.doc]

Supplementary Table 1. Diagnoses of patients presenting to secondary care with neither European Society of Cardiology guidelines based diagnosis of heart failure with preserved ejection fraction (HFpEF) or heart failure with reduced ejection fraction (HFrEF).

|  | **All**  n= 182 |
| --- | --- |
| **Diagnosis** |  |
| Angina/Ischaemic heart disease | 10 (5%) |
| Atrial fibrillation | 10 (5%) |
| Chest infection | 3 (2%) |
| Chronic obstructive pulmonary disease | 23 (13%) |
| Deconditioning | 5 (3%) |
| Dependant oedema | 6 (3%) |
| Diabetes mellitus | 2 (1%) |
| Hypertrophic obstructive cardiomyopathy | 2 (1%) |
| Hypertension | 26 (14%) |
| Lung cancer | 2 (1%) |
| No cause identified | 30 (16%) |
| Obesity | 10 (5%) |
| Right heart failure/Pulmonary hypertension | 42 (23%) |
| Valvular heart disease | 11 (6%) |

Data presented as n (%).
