# Supplementary material for: Atrial fibrillation and risk of progressive heart failure in patients with preserved ejection fraction heart failure
Source: ESC Heart Fail. 2022 Jul 4;9(5):3254–63. doi: 10.1002/ehf2.14004 (PMC9715884; doi:10.1002/ehf2.14004)
Supplement: Supplementary file 2 — Figure S1. Long term outcomes of patients with either heart failure with reduced (HFrEF) or preserved (HFpEF) left ventricular ejection fraction by tertiles of NTproBNP. Survival curves of death or hospitalization from progressive heart failure showing for tertiles of NTproBNP in those with (A) heart failure with preserved ejection fraction and (B) heart failure with reduced ejection fraction. [file EHF2-9-3254-s002.pptx]

## Slide 1
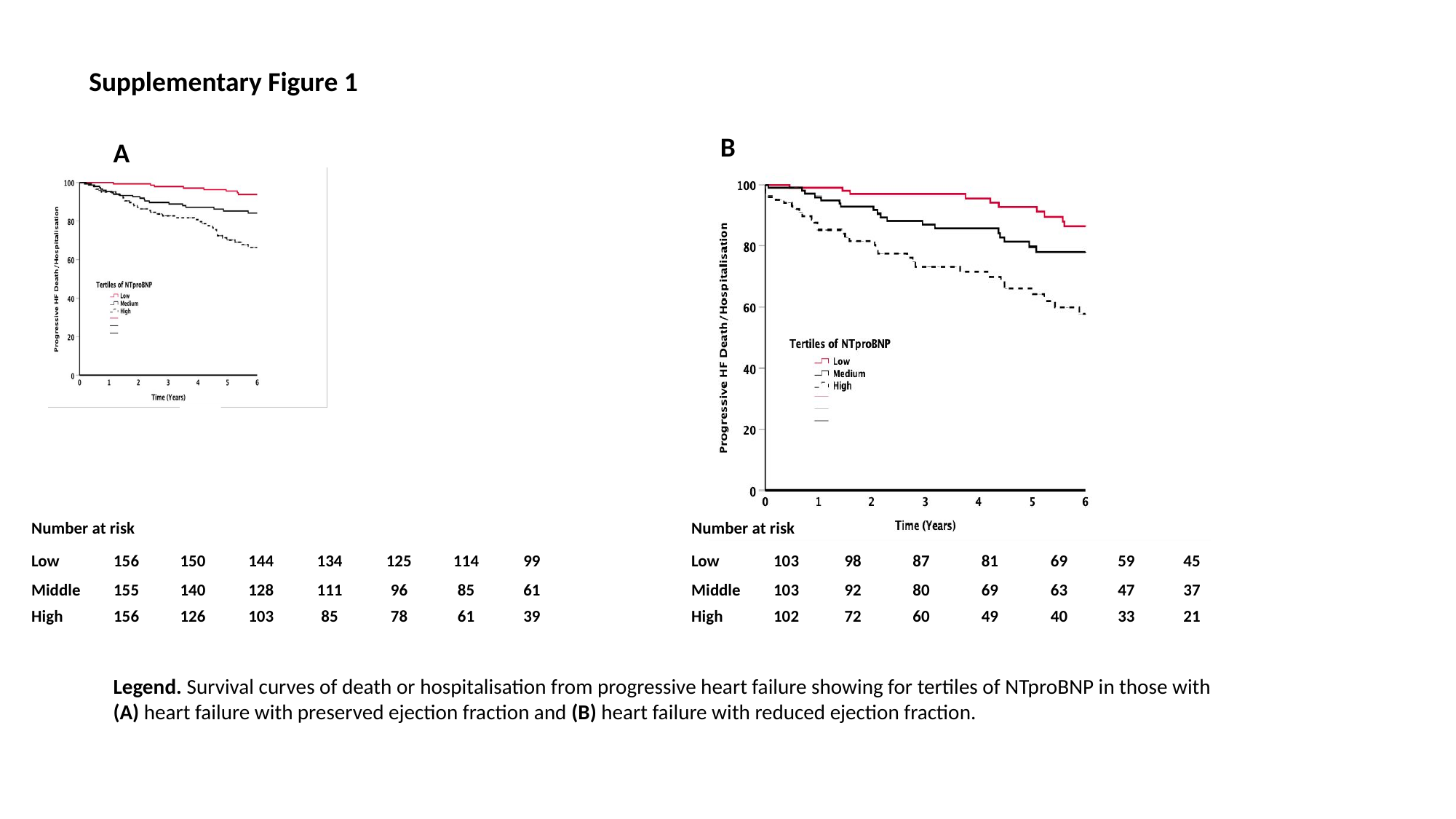

Supplementary Figure 1
B
A
| Number at risk | | | | | | | |
| --- | --- | --- | --- | --- | --- | --- | --- |
| Low | 156 | 150 | 144 | 134 | 125 | 114 | 99 |
| Middle | 155 | 140 | 128 | 111 | 96 | 85 | 61 |
| High | 156 | 126 | 103 | 85 | 78 | 61 | 39 |
| Number at risk | | | | | | | |
| --- | --- | --- | --- | --- | --- | --- | --- |
| Low | 103 | 98 | 87 | 81 | 69 | 59 | 45 |
| Middle | 103 | 92 | 80 | 69 | 63 | 47 | 37 |
| High | 102 | 72 | 60 | 49 | 40 | 33 | 21 |
Legend. Survival curves of death or hospitalisation from progressive heart failure showing for tertiles of NTproBNP in those with (A) heart failure with preserved ejection fraction and (B) heart failure with reduced ejection fraction.
